# Global prevalence of polypharmacy among the COVID-19 patients: a comprehensive systematic review and meta-analysis of observational studies

**DOI:** 10.1186/s41182-022-00456-x

**Published:** 2022-08-31

**Authors:** Hooman Ghasemi, Niloofar Darvishi, Nader Salari, Amin Hosseinian-Far, Hakimeh Akbari, Masoud Mohammadi

**Affiliations:** 1 Student Research Committee, Kermanshah University of Medical Sciences, Kermanshah, Iran; 2 Department of Biostatistics, School of Health, Kermanshah University of Medical Sciences, Kermanshah, Iran; 3 Department of Business Systems and Operations, University of Northampton, Northampton, UK; 4 Cellular and Molecular Research Center, Gerash University of Medical Sciences, Gerash, Iran

**Keywords:** Polypharmacy, Prevalence, COVID-19, Meta-analysis, Increased morbidity and mortality

## Abstract

**Background:**

Polypharmacy has traditionally been defined in various texts as the use of 5 or more chronic drugs, the use of inappropriate drugs, or drugs that are not clinically authorized. The aim of this study was to evaluate the prevalence of polypharmacy among the COVID-19 patients, and the side effects, by systematic review and meta-analysis.

**Methods:**

This study was performed by systematic review method and in accordance with PRISMA 2020 criteria. The protocol in this work is registered in PROSPERO (CRD42021281552). Particular databases and repositories have been searched to identify and select relevant studies. The quality of articles was assessed based on the Newcastle–Ottawa Scale checklist. Heterogeneity of the studies was measured using the *I*^2^ test.

**Results:**

The results of meta-analysis showed that the prevalence of polypharmacy in 14 studies with a sample size of 189,870 patients with COVID-19 is 34.6% (95% CI: 29.6–40). Studies have shown that polypharmacy is associated with side effects, increased morbidity and mortality among patients with COVID-19. The results of meta-regression analysis reported that with increasing age of COVID-19 patients, the prevalence of polypharmacy increases (*p* < 0.05).

**Discussion:**

The most important strength of this study is the updated search to June 2022 and the use of all databases to increase the accuracy and sensitivity of the study. The most important limitation of this study is the lack of proper definition of polypharmacy in some studies and not mentioning the number of drugs used for patients in these studies.

**Conclusion:**

Polypharmacy is seen in many patients with COVID-19. Since there is no definitive cure for COVID-19, the multiplicity of drugs used to treat this disease can affect the severity of the disease and its side effects as a result of drug interactions. This highlights the importance of controlling and managing prescription drugs for patients with COVID-19.

## Background

COVID-19, originating from the novel severe acute respiratory syndrome coronavirus 2 (SARS-CoV-2), appeared in December 2019 and quickly became a global pandemic [1, 2]. Despite the rapid spread of the disease, among 81% of patients, symptoms are mild and are usually treated at home [3]. Clinical manifestations of the disease range from asymptomatic to severe respiratory failure and death [4]. The most common symptoms of COVID-19 are fever and cough that occurs along with other symptoms such as dyspnoea, headache, muscle soreness, and fatigue [5, 6]. Although various drugs have been proven to treat COVID-19 disease in various clinical studies, there is no antiviral treatment with proven efficacy for COVID-19 patients [7].

Estimates show that more than 65% of adults over the age of 70 are at risk of severe COVID-19 infection [8]. A wide range of factors have been identified that affect the prognosis of COVID-19, including age, sex, ethnicity, and physical factors such as weight, body mass index, long-term conditions such as blood pressure, diabetes and stress [2]. Another case that is known as a health threat in particular among the older patients is polypharmacy [9].

Polypharmacy has traditionally been defined in various texts as the use of five or more chronic drugs, the use of inappropriate drugs, or drugs that are not clinically authorized [10]. Polypharmacy includes not only prescription drugs, but also over-the-counter and herbal medicines [11]. Polypharmacy often occurs among the elderly [12]. Polypharmacy in the elderly is a global problem that has recently worsened [13]. prevalence of polypharmacy ranges from 4 to 96.5% among community-dwelling older people to in hospitalized older people patients [14].

Polypharmacy due to its association with adverse health outcomes, including falls, functional impairment, drug adverse reactions, increased length of hospital stay, readmission, and mortality, is one of the important healthcare issues [12]. Numerous factors associated with polypharmacy, such as drug–drug interactions, drug–disease interactions, or potentially inappropriate prescriptions, may be involved in these adverse outcomes [12–14]. Polypharmacy is also a major factor in causing drug side effects before, during and after COVID-19 treatments [9]. In other words, polypharmacy can increase the risk of adverse drug events during COVID-19 treatment [7–9]. This may indicate that medications that may be helpful in treatment are not only not helpful in the event of drug side effects, but also delay treatment [7–9].

A systematic review by Iloanusi et al. [8] on the effect of polypharmacy on clinical outcomes in patients with coronavirus 2019 (COVID-19) has been performed and no meta-analysis has been performed to evaluate the overall prevalence of polypharmacy and therefore this study can improve it. .

Given the above, effective drug management is very important for treating COVID-19 patients. The aim of this study was to investigate the prevalence of polypharmacy in patients with COVID-19, using a systematic review and meta-analysis.

## Methods

### PROSPERO

This protocol has been registered in the Prospective Registry of Systematic Review database (CRD42021281552).

### Study approach and research question

The present systematic review and meta-analysis was conducted in accordance with the Preferred Reporting Items for Systematic Reviews and Meta-Analyses (PRISMA2020) and Cochran review approach. The stages in the systematic review process include: selecting a research question, determining inclusion and exclusion criteria, identifying articles, selecting studies, evaluating study quality, extracting data, and analysing and interpreting findings [15].

Our study aimed to answer the following research question “What is the global prevalence of polypharmacy among COVID-19 patients?” The study population (Population) includes: patients with COVID-19 worldwide, Outcome include: Prevalence of polypharmacy in patients with COVID-19, Time period duration for the search includes: no lower time limit and until June 22, 2022, and study type (study design) includes: observational (case control, cohort, cross sectional).

### Inclusion and exclusion criteria

Observational studies (case–control, cohort, cross-sectional) that have examined the prevalence of polypharmacy in patients with COVID-19 have been published in English, and their full text which was available and also includes the information in Table 1, study type, prevalence, mean age and sample size were eligible for inclusion in the study. Intervention and clinical trial, reviews including systematic review and meta-analysis were excluded.

**Table 1:** Search strategy for each database

Database	Search strategy	Date	Number of publications
PubMed	#1: (((((COVID-19[MeSH Terms]) OR ("SARS-CoV-2 Infection")) OR ("2019 Novel Coronavirus Infection")) OR ("2019 nCoV Disease")) OR ("Coronavirus Disease 2019")) OR (Coronavirus) #2: Polypharmacy [MeSH Terms] #3: (((Polypharmacy) OR (poly medication)) OR ("multiple drugs")) OR ("Potentially inappropriate medications") #4: (((((Outcome) OR (Mortality)) OR (death)) OR (morbidity)) OR (complication)) OR ("drug interactions") #5: #1 AND (#2 OR #3) AND #4	2022.6.22	143
Web of science	#1: ALL = (Polypharmacy OR polymedication OR "multiple drug" OR "Potentially inappropriate medications") #2: ALL = (Outcome OR Mortality OR death OR morbidity OR complication OR "drug interactions") #3: TS = ("COVID-19" OR "SARS-CoV-2 Infection" OR "2019 Novel Coronavirus Infection" OR "2019 nCoV Disease" OR "Coronavirus Disease 2019" OR Coronavirus) #4: #1 AND #2 AND #3	2021.6.22	68
Scopus	#1: TITLE-ABS-KEY ("COVID-19" OR "SARS-CoV-2 Infection" OR "2019 Novel Coronavirus Infection" OR "2019 nCoV Disease" OR "Coronavirus Disease 2019" OR coronavirus) #2: ALL (outcome OR mortality OR death OR morbidity OR complication OR "drug interactions") #3: TITLE-ABS-KEY ((polypharmacy OR polymedication OR "multiple drug" OR "Potentially inappropriate medications") #4: #1 AND #2 AND #3	2021.6.22	252
Embase	#1: ‘covid 19’:ti,ab,kw OR ‘sars-cov-2 infection’:ti,ab,kw OR ‘2019 novel coronavirus infection’:ti,ab,kw OR ‘2019 ncov disease’:ab,kw OR 'coronavirus disease 2019':ti,ab,kw OR coronavirus:ti,ab,kw #2: polypharmacy:de #3: polypharmacy:ti,ab,kw OR polymedication:ti,ab,kw OR 'multiple drug':ti,ab,kw OR 'potentially inappropriate medications':ti,ab,kw #4: outcomes:ti,ab,kw OR mortality:ti,ab,kw OR death:ti,ab,kw OR morbidity:ti,ab,kw OR complication:ti,ab,kw OR 'drug interactions':ti,ab,kw #5: #2 OR #3 #6: #1 AND #4 AND #5	2021.6.20	138
ScienceDirect	Title, abstract or author-specified keywords (COVID-19 OR "sars-cov-2") AND (Polypharmacy OR polymedication OR "multiple drug" OR "Potentially inappropriate medications")	2021.6.22	38
ProQuest	#1: (Polypharmacy OR polymedication OR "multiple drug" OR "Potentially inappropriate medications") #2: (Outcome OR Mortality OR death OR morbidity OR complication OR "drug interactions") #3: TI,AB("COVID-19" OR "SARS-CoV-2 Infection" OR "2019 Novel Coronavirus Infection" OR "2019 nCoV Disease" OR "Coronavirus Disease 2019" OR Coronavirus) #4: #1 AND #2 AND #3	2021.6.22	389

### Search strategy, and article identification

Systematic search of documents in international databases was performed with selected keywords. The search process was carried out for ScienceDirect, Web of Science (WoS), ProQuest, Embase, Medline (PubMed), and Scopus reference management databases. The Google Scholar search engine was also searched to ensure the comprehensiveness of the search process. Gray Literature, i.e. studies that their results have not been published were also examined within related databases and also by searching the reference lists of identified studies.

Keywords were extracted from the Medical Subject Headings (MeSH) database. Keywords related to the studied population (P) were: COVID-19, SARS-CoV-2, 2019-ncov infection and outcome-related keywords (O) were: polypharmacy, drug interaction, potentially inappropriate medications, mortality, morbidity, outcomes based on Mesh browser. The search strategy in each database was determined by using the Advanced Search option and using all possible keyword combinations with the help of AND, and OR operators (Table1). The characteristics of the extracted studies are listed in Table 2.

**Table 2:** Characteristics of the selected studies

First author	Year of publication	Country	Definition of polypharmacy	Study design	Participants	Mean age (SD)	Patients with poly pharmacy
Bağ Soytaş [19]	2021	Turkey	≥ 5	Retrospective study	218	75.3	108
Bayrak [20]	2022	Turkey	≥ 5	Prospective study	122	73	59
Cantudo-Cuenca [21]	2021	Spain	≥ 5	observational study	174	67	92
Carrillo-Garcia [22]	2021	Spain	≥ 5	Longitudinal study	165	88.5	112
Couderc [23]	2021	France	≥ 5	Retrospective study	480	88	348
Crescioli [17]	2021	Italy	> 5	Case series	23	76.1 (14.40)	16
De Smet [24]	2020	Belgium	≥ 5	Retrospective study	81	85	52
Gavin et al. [25]	2020	America	≥ 5	Retrospective chart review	140	60	NR
Kananen et al. [26]	2021	Sweden	≥ 5	observational study	1409	83(12)	NR
Klanidhi [27]	2022	India	≥ 5	Prospective study	60	68.76	23
Laosa [28]	2020	Spain	≥ 5	Prospective study	375	66.06	77
Lim et al. [29]	2021	Singapore	≥ 4	Observational study	275	59 (54–66)	73
Lozano-Montoya [30]	2021	Spain	≥ 5	Longitudinal study	300	86.3	213
Manjhi [31]	2021	India	≥ 5	Retrospective study	200	> 40	142
Mannucci [18]	2022	Italy	≥ 5	Observational study	48,148	NR	7464
McKeigue et al. [32]	2021	Scotland	≥ 5	Matched case control	4251	0–75, ≥ 75	NR
McQueenie et al. 1 [2]	2020	England	4–6	Retrospective study	1324	48–86	298
McQueenie et al. 2 [2]	2020	England	6–9	Retrospective study	1324	48–86	130
McQueenie et al. 3 [2]	2020	England	> 10	Retrospective study	1324	48–86	72
Poblador-Plou [33]	2020	Spain	≥ 5	Retrospective study	4412	67.7	1429
Rodriguez-Sanchez [34]	2021	Spain	5_9	Cohort study	499	86.7	200
Rodriguez-Sanchez [34]	2021	Spain	> 10	Cohort study	499	86.7	163
Sirois 1 [35]	2022	Canada	5–9	Population-based study	32,476	79.59	9579
Sirois 2 [35]	2022	Canada	10–14	Population-based study	32,476	79.59	8619
Sirois 3 [35]	2022	Canada	15–19	Population-based study	32,476	79.59	5009
Sirois 4 [35]	2022	Canada	≥ 20	Population-based study	32,476	79.59	3746
Sun et al. [36]	2020	China	≥ 5	Retrospective study	217	45.7 (16.6)	NR
Taher [37]	2020	Bahrain	≥ 5	Retrospective study	73	54	43

In order to access the latest published studies, an alert was created on a number of important databases, including PubMed and Scopus, to check if any new article was published during the study. Also, in order to access all related studies, the sources of articles that met the inclusion criteria were manually reviewed. To avoid errors and mistakes, all steps of article search, study selection, quality evaluation and data extraction were performed by two reviewers (researchers) independently. For this purpose, the information of all articles found in each database was transferred into the EndNote X8 references management software. After completing the search in all the databases, duplicate articles were removed. If there was a difference of opinion between the researchers regarding the inclusion of the article in the study, in order to avoid the risk of bias for specific studies, a final agreement was reached first through discussion and in some cases with the participation and opinion of a third reviewer.

### Quality evaluation of observational studies

The quality of articles was assessed based on selected and related items of the Newcastle–Ottawa Scale (NOS) checklist. The items on the checklist include: study design, background, place and time of study, outcome, inclusion criteria, sample size and statistical analysis. The NOS sets a maximum of 9 points for the lowest risk of bias in three areas: four points for selection of study groups; (2) two points for comparison of groups and three points for determining the amount of exposure and results for case and group studies. Based on this, we considered articles with a score of 7 and above as high-quality articles [16].

### Data extraction

After selecting the studies for the systematic review and meta-analysis process, the data were extracted, and the studies were summarized. An electronic checklist was prepared for this purpose. The items in the checklist include: surname of the first author, year of publication and year of study, place of study, age, sample size, total number of people with COVID-19, number of people with polypharmacy. Other distinct checklists were used to extract data different sections: one checklist was designed to extract statistical data (for meta-analysis), one checklist was designed to extract characteristics of the articles (for complete review and also for component analysis). Also, to increase the accuracy of the work, articles that the target population with an underlying disease, and articles that the study population did not have a specific underlying disease, were separated and extracted with different checklists and then patients with underlying disease were excluded from the study.

### Statistical analysis

To analyse and combine the results of different studies, in each study, data about treatment methods and other small values were considered as binominal probability and its variance was calculated through binominal distribution. Heterogeneity of studies was assessed using the *I*^2^ test. Publication bias was assessed the Egger’s test and corresponding funnel plots were drawn. Data were analysed within Comprehensive Meta-Analysis software (version 2).

## Results

**Fig. 1:**
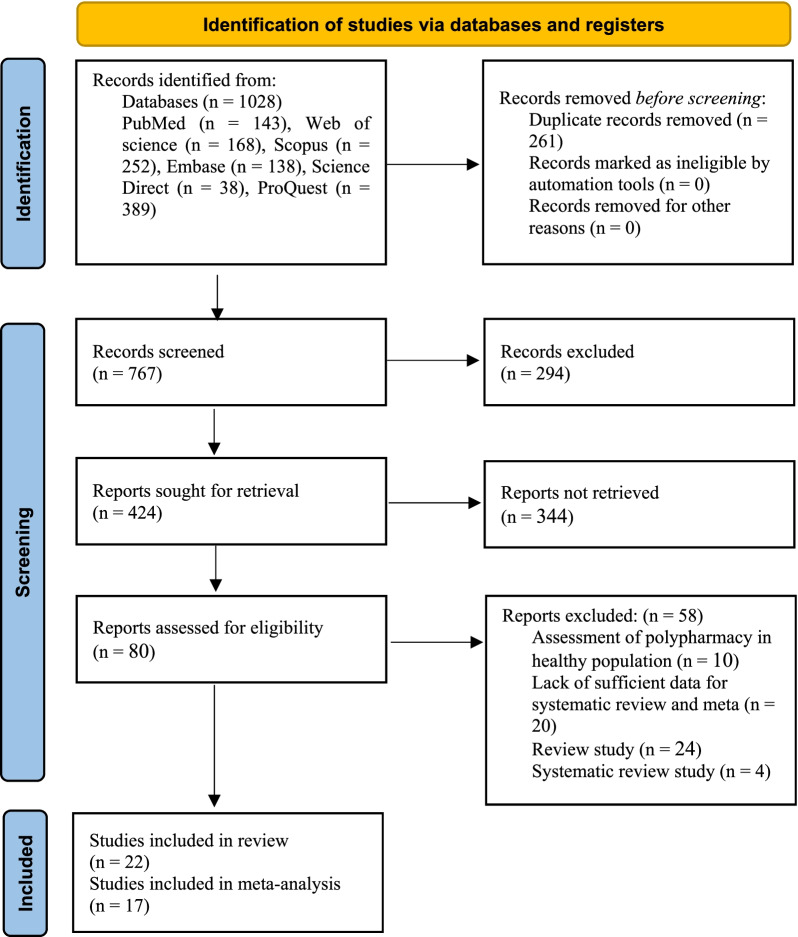
PRISMA flow diagram for study selection

After a systematic search of the specified databases, a total of 1028 articles were identified and entered into EndNote. After deleting 261 duplicate articles, the titles and abstracts of 767 articles were reviewed according to the inclusion and exclusion criteria, and 80 articles remained for the secondary evaluation and further review. At this stage, the full text of the articles was reviewed in accordance with the inclusion and exclusion criteria, and finally 22 articles were approved and entered the systematic review process (Fig. 1).

All studies were conducted in 2020 and 2022. Of the 22 confirmed studies, 14 were conducted in continental Europe. Of these studies, 5 were conducted in Spain. The other 9 were conducted in Belgium, Scotland, Italy, the United Kingdom, Turkey, France, and Sweden. Other studies were conducted in Asia and the Americas. Among these, 3 studies took place in Bahrain, China, India, and Singapore and another study was conducted in the United States, and Canada.

Among the studies performed, 17 pieces of research were retrospective, prospective, and longitudinal, one study, case series and the only remaining study being a case control. In these studies, a total of 95,422 people were studied. Least participant was in the study by Crescioli et al. [17] with 23 people and the most participants were reported in the study of Mannucci et al. [18] with 48,148 people (Table 2).

### Prevalence of polypharmacy in patients with COVID-19

**Fig. 2:**
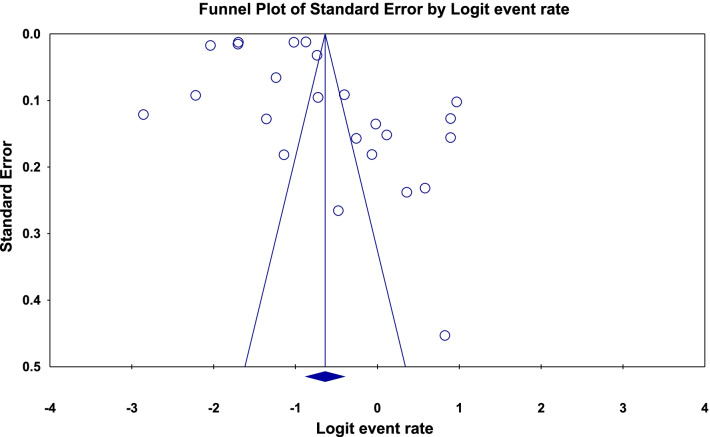
Funnel plot diagram on the publication bias among the studies

**Fig. 3:**
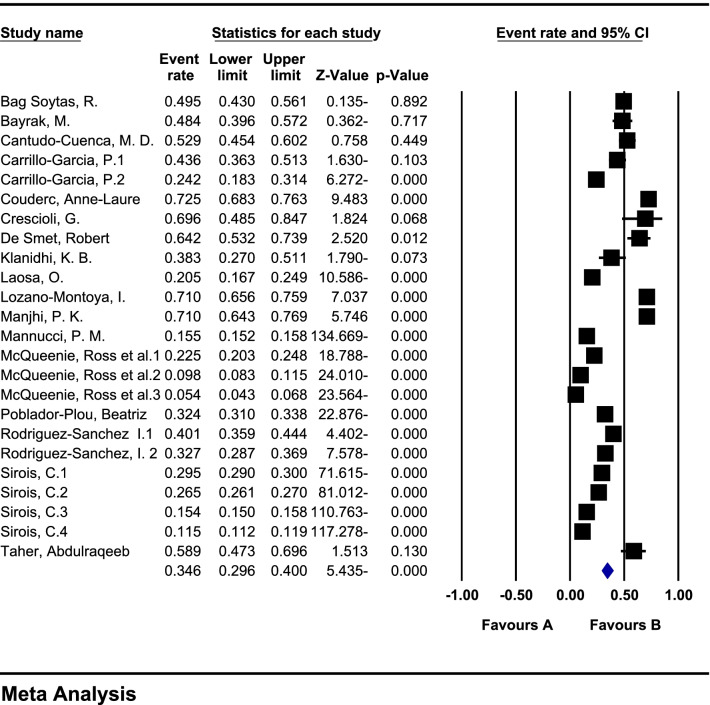
Forest plot and general meta-analysis of the results of studies based on random effects method

In a review of 17 studies with a sample size of 189,870 patients with COVID-19, the heterogeneity of the studies was evaluated based on the *I*^2^ test (*I*^2^: 99.6) and based on the high heterogeneity in the studies, the random effects method was used to analyse the studies. Publication bias was not significant in the studies (*p* = 0.183) (Fig. 2). The overall prevalence of polypharmacy in patients with COVID-19 based on meta-analysis was reported to be 34.6% (95% CI: 29.6–40) (Fig. 3).

**Fig. 4:**
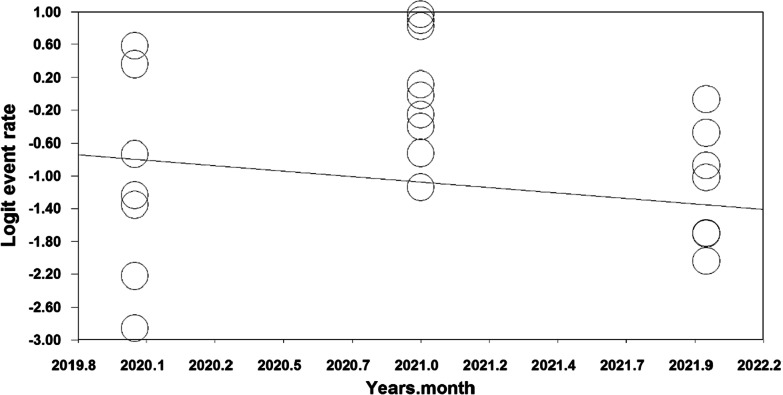
Meta-regression diagram of the prevalence of polypharmacy in patients with COVID-19 by year of study (month)

**Fig. 5:**
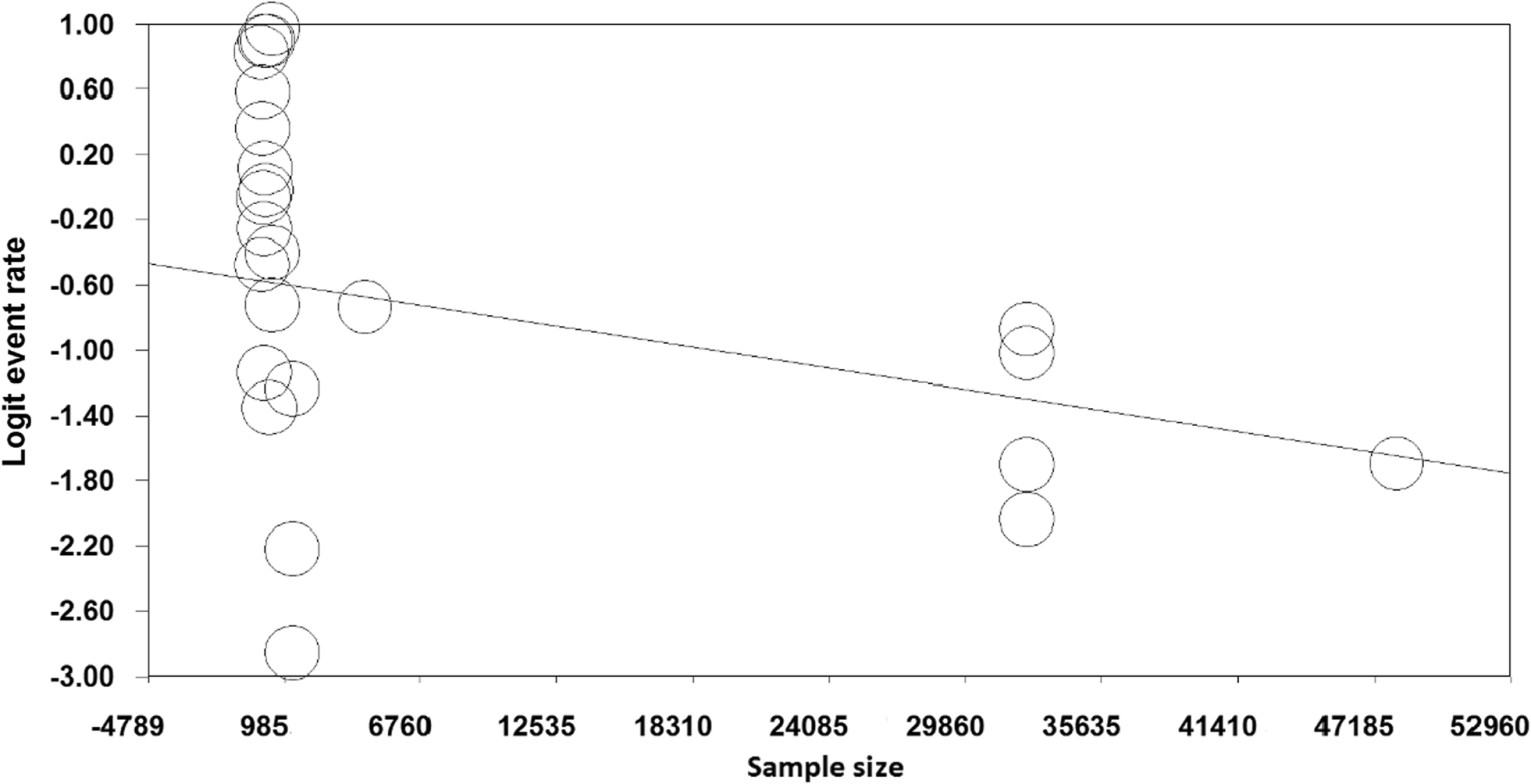
Meta-regression of the prevalence of polypharmacy in COVID-19 patients by sample size

**Fig. 6:**
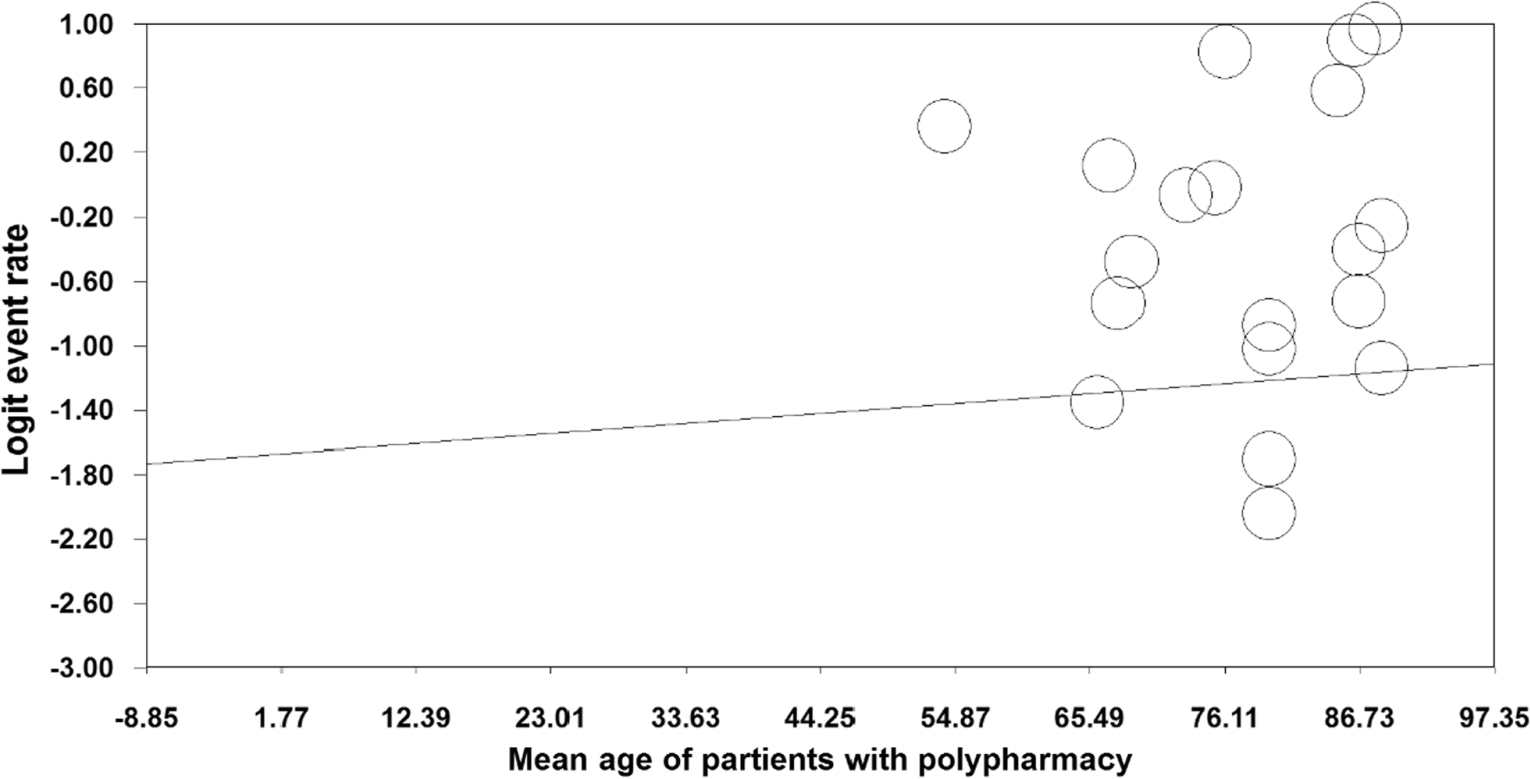
Meta-regression diagram of the prevalence of polypharmacy in patients with COVID-19 by age of study participants

The results of the meta-regression analysis based on sample size, year of study and age of study participants also showed that with increasing year of study (month), the prevalence of polypharmacy in COVID-19 patients decreased (*p* < 0.05) (Fig. 4), With increasing sample size, the prevalence of polypharmacy in COVID-19 patients decreased (*p* = 0.07) (Fig. 5). As the age of patients with COVID-19 increased, the prevalence of polypharmacy in patients with COVID-19 increased (*p* = 0.05) (Fig. 6).

In Table 3, subgroup analysis was performed based on the number of drugs as well as the patient’s condition after treatment, and it was reported that the highest prevalence of polypharmacy was in patients with COVID-19 treated with 4–9 drugs with a prevalence of 26.8 (95% CI: 18.5–37.1) and also polypharmacy in patients who did not survive after treatment with a prevalence of 54.8 (95% CI: 45.4–63.9) was higher (Table 3).

**Table 3:** Subgroup analysis of results by number of drugs and status of patients with COVID-19 after treatment

Subgroup		*N*	Sample size	Heterogeneity (*I*^2^)	Egger test	Prevalence (95% CI)
Polypharmacy by drug number	4–9	5	35,788	98.5	0.631	26.8 (95% CI: 18.5–37.1)
	> 10	6	99,416	99.8	0.751	17.1 (95% CI: 11.6–24.3)
Patient status after treatment	Survivors	9	5490	98.2	0.144	42.1 (95% CI: 28.9–56.6)
	Non-survivors	9	1508	88.3	0.972	54.8 (95% CI: 45.4–63.9)

### Association of polypharmacy with increasing disease severity and mortality

Polypharmacy is a common issue among the elderly. This problem was also observed in patients with COVID-19, as the study of Sun et al. [36] reported that with increasing age, the drugs used in patients with COVID-19 also increased. Studies have shown that polypharmacy is associated with increased side effects. A study by Taher et al. [37] showed that an increase in polypharmacy is associated with an increase in acute kidney injury. McQueenie et al. [2] study also showed that polypharmacy in patients with COVID-19 was associated with an increase in disease severity, which was statistically significant. This result was also observed in the study of McKeigue et al. [32].

The study by Lim et al. [29] also emphasized that the prevalence of polypharmacy increases with the age of patients with COVID-19. In the study by Mannucci et al. [18] showed that polypharmacy was lower in patients at home than in hospitalized patients. Patients admitted to the intensive care unit (ICU) showed the highest exposure to polypharmacy. On the other hand, it was observed that the number of patients exposed to polypharmacy in the ICU during the corona pandemic was significantly higher than this amount before the outbreak. The study by Sirois et al. [35] also showed that polypharmacy increases the risk of hospitalization and even mortality from the disease.

The relationship between polypharmacy and increased mortality has also been investigated in some studies. According to the results of 6 studies [19, 20, 28, 33–35], with increasing polypharmacy in the selected samples, mortality due to COVID-19 also increases, which is statistically significant. However, in 2 other studies [26, 30], it was stated that the increase in medications used did not have a significant effect on the increase in mortality caused by COVID-19.

Only in one study [22] was it observed that polymedication for the control and treatment of diseases that existed before the onset of COVID-19 had a protective effect on mortality and reduced mortality due to COVID-19.

In addition, the study by Gavin et al. [25] reported that polypharmacy had no significant relationship with the need for ventilator respiratory support. In this study, there was no statistical difference between people who improved after connecting to a ventilator and people who died after connecting to a ventilator.

## Discussion

The present study was conducted for the first time with the aim of investigating the prevalence of polypharmacy among patients with COVID-19 globally, by a systematic review. The results of this study showed that 34.6% of patients with COVID-19 had polypharmacy. The results of meta-regression also showed that with the increase of the study year, polypharmacy studies decreased. Also, based on the sample size of the study, meta-regression indicated that the prevalence of polypharmacy decreased with the increase in the number of participants in the study and additionally, as the age of patients with COVID-19 increased, the prevalence of polypharmacy in patients with COVID-19 increased. This may be due to the nature of COVID-19 disease and its greater impact on the elderly, which requires the use of more drugs to treat COVID-19 in the elderly.

In the case of prescribing inappropriate medicine for the person, the incidence of side effects associated with the use of inappropriate drugs increases [38]. In other words, it seems necessary to maintain the quality of life of some elderly impacted by polypharmacy, however inappropriate drugs may be associated with side effects that increase the burden of disease among the elderly [39].

The most vulnerable patients to COVID-19 are the elderly and patients with underlying problems such as high blood pressure, diabetes, cardiovascular disease, chronic respiratory disease, and cancer. These patients are typically exposed to a large number of medications during the day. A study by Al Rihani et al. looked at the elderly with underlying diseases, and found that participants in the study took an average of about 11 different medications during the day. This increases the incidence of drug interactions and adverse drug events. In addition to being more prone to COVID-19, these patients are more likely to experience associated side effects. However, the risk of using any of the COVID-19 recommended drugs in such elderly people with polypharmacy is still high, yet no previous study on the concept has been conducted [40].

Along with the COVID-19 pandemic, and the increase in the incidence of this disease, polypharmacy increased in patients with COVID-19, especially in older population. This is justified by the fact that there is no definitive cure for the disease. Also, the widespread side effects of COVID-19 increase the need for symptomatic treatments in individuals, which is also effective in increasing the use of various drugs. Other studies have confirmed the rise in polypharmacy in adults with COVID-19. A study by Nwanaji-Enwerem et al. Reported that in Africa, polypharmacy is a growing health threat as the population ages and the prevalence of several diseases increases, the effects of polypharmacy in Africa can be mitigated by strengthening training in evidence-based prescribing and joint decision-making [41]. Research works in previous epidemics have also shown that patients with polypharmacy are more likely to develop the disease and increase side effects [42, 43].

Studies in this systematic review and meta-analysis have shown that polypharmacy is associated with an increase in adverse side effects such as acute kidney injury, adverse drug reaction, increased severity of COVID-19 and increased mortality due to this disease [8]. With increasing polypharmacy, the incidence of drug interactions in patients also increases. One study found that COVID-19 patients admitted to medical facilities were at high risk for drug interactions. Treatments to control infection in these patients, including concomitant treatment with lopinavir/ritonavir and hydroxychloroquine, significantly increase drug interactions [44]. As the complexity of medication regimens increases, so does the pressure on healthcare systems. Medication errors with inappropriate drugs significantly increase the risk of adverse consequences. The incidence of aging syndromes, including falls and delirium, previously exacerbated by polypharmacy, is accelerated by COVID-19 treatments. And their management will be more difficult because infection control is a priority in patients with COVID-19 [45]. Therefore, physicians should manage the risk of drug interactions when prescribing new drugs to treat and control the symptoms of COVID-19 [46].

### Strength and limitations

The most important strength of this study is the updated search to June 2022 and the use of all databases to increase the accuracy and sensitivity of the study, and the most important limitation of this study is the lack of proper definition of polypharmacy in some studies and not mentioning the number of drugs used for patients in these studies.

## Conclusion

The results of this study showed that polypharmacy is highly prevalent among patients with COVID-19, especially among the elderly. It has also been observed that adverse outcomes such as renal problems, drug interactions, increased risk and severity of COVID-19, and increased mortality in people with polypharmacy are more common than others.

## Data Availability

Not applicable.

## Abbreviations

SARS-CoV-2: Severe acute respiratory syndrome coronavirus 2
MESH: Medical Subject Headings
WoS: Web of Science
PRISMA: Preferred Reporting Items for Systematic Reviews and Meta-Analysis
NOS: Newcastle–Ottawa Scale
